# Association of peripheral basophils with tumor M2 macrophage infiltration and outcomes of the anti-PD-1 inhibitor plus chemotherapy combination in advanced gastric cancer

**DOI:** 10.1186/s12967-022-03598-y

**Published:** 2022-09-04

**Authors:** Chaorui Wu, Yaopeng Qiu, Renyi Zhang, Xiaoqing Li, Huayuan Liang, Minghao Wang, Fengping Li, Mansheng Zhu, Gengtai Ye, Hao Liu, Guoxin Li, Liying Zhao

**Affiliations:** 1 Department of General Surgery & Guangdong Provincial Key Laboratory of Precision Medicine for Gastrointestinal Tumor, Nanfang Hospital, The First School of Clinical Medicine, Southern Medical University, Guangzhou, Guangdong 510515 China; 2 Department of Pathology, Nanfang Hospital, Southern Medical University, Guangzhou, Guangdong China

**Keywords:** Gastric cancer, Anti-programmed death-1, Peripheral blood, Basophils, M2 macrophages

## Abstract

**Background:**

Although the anti-programmed death-1 (PD-1) inhibitor plus chemotherapy combination has been approved as the standard first-line treatment for advanced gastric cancer, a proportion of patients do not significantly benefit from this therapy. Who would respond poorly to this treatment and the underlying mechanisms of treatment failure are far from clear.

**Methods:**

We retrospectively analyzed the associations between the peripheral basophils at baseline and clinical outcomes in 63 advanced gastric cancer patients treated with anti-PD-1 plus chemotherapy and 54 patients treated with chemotherapy alone. Immunohistochemistry and immunofluorescence staining in gastric cancer samples were utilized to investigate the basophil-related immunophenotype.

**Results:**

The optimal cutoff of basophil count to distinguish responders to anti-PD-1 plus chemotherapy from non-responders was 20.0/μL. Compared with the low basophil group (≤ 20.0/μL, n = 40), the high basophil group (> 20.0/μL, n = 23) had a significantly lower objective response rate (ORR 17.4% vs. 67.5%, p = 0.0001), worse progression-free survival (median PFS 4.0 vs. 15.0 months, p = 0.0003), and worse overall survival (median OS not reached, p = 0.027). Multivariate analyses identified a basophil count of > 20.0/μL as an independent risk factor for a worse ORR (OR 0.040, 95% CI 0.007–0.241, p = 0.0004), worse PFS (HR 3.720, 95% CI 1.823–7.594, p = 0.0003) and worse OS (HR 3.427, 95% CI 1.698–6.917, p = 0.001). In contrast, there was no significant association between peripheral basophil counts and tumor response or survival in the chemotherapy-alone group (p > 0.05). In primary gastric cancer samples, we observed a correlation between higher peripheral basophil counts and the accumulation of tumor-infiltrating basophils (r = 0.6833, p = 0.005). Tumor-infiltrating basophils were found to be spatially proximate to M2 macrophages within TME and positively correlated with tumor M2 macrophage infiltration (r = 0.7234, p = 0.0023). The peripheral basophil counts also had a significant positive correlation with tumor-infiltrating M2 macrophage counts (r = 0.6584, p = 0.003). Further validation in tumor samples treated with the neoadjuvant anti-PD-1 inhibitor plus chemotherapy combination suggests that the peripheral basophils, tumor infiltration of basophils, and M2 macrophages were significantly more abundant in non-responders than in responders (p = 0.0333, p = 0.0007, and p = 0.0066, respectively).

**Conclusions:**

The peripheral basophil count was observed to be a potential biomarker of anti-PD-1 efficacy for advanced gastric cancer. Moreover, basophils may induce an immune-evasive tumor microenvironment by increasing M2 macrophage infiltration, which could be a potential immunotherapeutic target for advanced gastric cancer.

**Supplementary Information:**

The online version contains supplementary material available at 10.1186/s12967-022-03598-y.

## Introduction

Gastric cancer remains one of the most aggressive types of human cancer, ranking among the five leading causes of cancer incidence and mortality worldwide [1, 2]. The anti-programmed death 1 (PD-1) inhibitor plus chemotherapy combination showed satisfactory toxicity and antitumor activity for advanced gastric cancer according to the results of the CheckMate 649 trial, with an objective response rate of approximately 60% and a median overall survival of 14.3 months [3]. Nevertheless, the clinical benefit is limited for a significant portion of patients, and treatment resistance frequently occurs [4]. Immunotherapy can also be associated with immune-related adverse events and high treatment costs. Hence, identifying patients who will benefit from the anti-PD-1 inhibitor plus chemotherapy combination remains a priority.

The biomarkers predictive of anti-PD-1 response in gastric cancer at this stage include PD-L1 expression, the microsatellite instability (MSI)/mismatch repair (MMR) status, Epstein-Barr virus (EBV) infection, and the tumor mutation burden (TMB) [5, 6]. These biomarkers only focus on the inherent features of tumor cells and miss interactions with other tumor microenvironment (TME) components [7], which partially accounts for the unsatisfactory predictive efficacy of the anti-PD-1 response [8–10]. As the most abundant cell component in TME, tumor-associated macrophages (TAMs) typically present with an M2-like phenotype [11] that is well known to induce immunosuppression through various mechanisms [12–14], which promotes cancer progression [15, 16] and resistance to immunotherapy [17].

Although tumor biopsy is widely adopted for immunotherapy biomarker identification and characterization, it is challenging to obtain tissues because of limited accessibility and invasiveness, especially for advanced disease, which also hinders the accessibility of these biomarkers. Emerging evidence suggests that the localized antitumor immune response has to be sustained by the continuous communication between the TME and the peripheral blood [18, 19]. Hence, peripheral blood analyses have the potential for TME immune monitoring. It is vital to develop biomarkers that could help monitor the status of M2 macrophages within the TME through peripheral blood analyses which are thought to be more readily accessible and noninvasive.

In this study, we analyzed the association of all peripheral leukocyte subpopulations prior to treatment with clinical outcomes in advanced gastric cancer. We also performed immunofluorescence (IF) and immunohistochemistry (IHC) evaluations of tumor samples to characterize the basophil-related TME phenotype.

## Materials and methods

### Study population

We retrospectively analyzed data collected at a tertiary hospital (Guangzhou, China) between November 2019 and December 2021. Patients were included if they had recurrent or metastatic gastric cancer and were treated with the anti-PD-1 inhibitor plus chemotherapy combination. The study population also comprised a control group consisting of patients with advanced gastric cancer treated with chemotherapy alone in the same period. Patients were eligible if they had received at least three cycles of treatment, had measurable (at least one lesion) or evaluable disease per the Response Evaluation Criteria in Solid Tumors (RECIST, version 1.1), underwent peripheral blood examination within 2 weeks before treatment initiation and prior to cycle 3, and underwent regular radiological assessments. Patients were excluded if they had a concomitant hematological malignancy, recent infection or inflammation, allergic diseases, or if they received any other anti-tumor therapy within one week prior to blood sampling for the basophil count assessment.

The most recent complete blood counts obtained before the initiation of cycle 1 (up to two weeks before the first treatment) and prior to cycle 3 were retrieved from electronic medical records. Patients were treated until the occurrence of disease progression, intolerable toxicity, and/or the doctors’ decision to discontinue treatment. Contrast-enhanced CT, MRI, or PET-CT at baseline, at week 12, and every 12 weeks thereafter were conducted to assess tumor response. Clinical responses were determined according to RECIST 1.1 and classified as complete response (CR), partial response (PR), stable disease (SD), and progressive disease (PD). Depending on the best overall response, patients were classified as non-responders (SD or PD) and responders (CR or PR). A separate cohort of gastric cancer patients who underwent surgery without any preoperative therapy, as well as patients treated with neoadjuvant anti-PD-1 plus chemotherapy and surgery, were included to validate findings in tumor samples.

The study protocol complied with the principles defined in the Declaration of Helsinki. Written informed consent was given by all the patients in this study before treatment. All blood tests and treatments were performed in accordance with relevant clinical guidelines.

### Treatment regimens

PD-1-targeting antibodies included Nivolumab, Pembrolizumab, Sintilimab, Camrelizumab, or Toripalimab every three weeks. Chemotherapy regimens involved XELOX (capecitabine plus oxaliplatin), SOX (S-1 plus oxaliplatin), FOLFOX (leucovorin, fluorouracil, and oxaliplatin), FLOT (fluorouracil, leucovorin, oxaliplatin, and docetaxel), DCF (docetaxel plus fluorouracil), and other combinations. The regimen was based on the patient’s condition and preference.

### Assessment of PD-L1 expression

The PD-L1 combined positive score (CPS) was determined by immunohistochemistry using validated anti-PD-L1 antibodies: E1L3N (Cell Signaling Technology, Danvers, Massachusetts, USA) and 22C3 (Dako North America Inc, Carpinteria, California, USA).

### Assessment of the MMR status

The mismatch repair (MMR) status was routinely assessed by immunohistochemistry (IHC) staining of four proteins (MLH1, PMS2, MSH2, and MSH6). Tumors with a deficient MMR (dMMR) phenotype were defined as showing a loss of expression of one or more MMR proteins. A proficient MMR (pMMR) phenotype was defined as one showing intact MMR protein expression.

### Assessment of the human epidermal growth factor receptor 2 (HER2) expression status

The human HER2 status should be tested by IHC and/or the FISH test. HER2 positivity is defined as the number of tumor cells showing a strong overexpression (3+) exceeding 10% of the total tumor population. If the number of tumor cells displaying moderate HER2 overexpression (2+) exceeds 10% of the total tumor population, the HER2 status is equivocal and negative otherwise. If the initial HER2 result is equivocal by IHC, then a FISH assay should be added to confirm the HER2 status.

### Assessment of the EBV infection status

EBV infection was detected by chromogenic in situ hybridization with EBV-encoded small RNA (EBER) using fluorescein-labeled oligonucleotide probes. The positive EBER nuclear expression in tumor cells with negative signals in normal tissues was EBV-positive.

### Scoring pathologic response

The tumor regression of gastric cancer after neoadjuvant anti-PD-1 therapy was graded according to a scoring system developed by the American Joint Committee on Cancer to measure the response of rectal cancer to neoadjuvant chemoradiation. The pathologic response ranges from 0 to 3 as follows: 0 (complete response), no viable cancer cells; 1 (marked response), single or small groups of cancer cells; 2 (moderate response), residual cancer outgrown by fibrosis; and 3 (poor or no response), minimal or no tumor kill, extensive residual cancer, or tumor progression. Tumor regression of grades 0–1 was considered as a pathologic response and the remaining were defined as no pathologic response.

### Hematoxylin–eosin, immunofluorescence, and immunohistochemistry staining

Detailed information is presented in Additional file 1. Tumor samples were collected from gastric cancer patients who did not receive any preoperative therapy and patients receiving neoadjuvant anti-PD-1 therapy and curative surgery. All staining was conducted on sections of formalin-fixed paraffin-embedded tumor tissues. Basophils were stained using anti-Pro Major Basic Protein 1 (ProMBP1) antibodies (Biolegend, San Diego, USA, catalog number: 346802). M2 macrophages were assessed using an anti-CD163 antibody (Abcam, Cambridge, UK, catalog number: ab182422). For IHC, the slides were incubated with peroxidase-conjugated secondary antibodies, stained using diaminobenzidine (DAB)-H_2_O_2_, and counterstained with hematoxylin. For IF, fluorescence-labeled secondary antibodies were used and DAPI was counterstained.

### Analysis of stained tumor samples

Detailed information is provided in Additional file 1. The IHC results were evaluated by two independent observers who were blinded to the clinical data. The mean number of stained basophils and CD163^+^ macrophages was counted in three different areas at 400 × magnification. Observed cell numbers were divided by the evaluated area to obtain an average cell density. Major discrepancies in cell counts were reviewed and reanalyzed together to reach a consensus.

### Statistical analysis

Overall survival (OS) was defined as the interval from the first dose of the anti-PD-1 inhibitor plus chemotherapy combination to either death from any cause or the last follow-up. Progression-free survival (PFS) was defined as the interval from the first dose of the anti-PD-1 inhibitor plus chemotherapy combination to disease progression documented by imaging, death, or last follow-up. The cutoff basophil counts for response (CR/PR) were determined using time-dependent receiver operating characteristic (ROC) analysis. Categorical variables were analyzed using the chi-square test or Fisher’s exact test, and variables from the peripheral blood were examined for normality of distribution and compared either with the Mann–Whitney U-test or the two-sample T-test. Kaplan–Meier survival curves were plotted and compared using the log-rank test. Factors associated with clinical response were explored using binary logistic regression analyses. Covariates associated with PFS and OS were evaluated through univariable and multivariate Cox regression analyses. Variables that reached statistical significance at p ≤ 0.1 were allowed to enter into the multivariate analyses. Correlations between two parameters were estimated using the Pearson correlation coefficient. All p-values were two-sided and confidence intervals (CI) were at the 95% level, with significance predefined to be at p < 0.05. All statistical tests were conducted using SPSS version 22.0 (SPSS Inc, Chicago, IL, USA) or GraphPad Prism (version 8.0e, GraphPad Software).

## Results

### Patient characteristics and outcomes

Between November 2019 and October 2021, 139 consecutive patients treated with the anti-PD-1 plus chemotherapy combination (the anti-PD-1 plus chemotherapy group) and 120 patients treated with chemotherapy alone (the chemotherapy-alone group) were initially included. Finally, 63 and 54 patients in the anti-PD-1 plus chemotherapy group and the chemotherapy-alone group, respectively, were enrolled in this study. The demographic profiles and disease characteristics of the patients in the two groups are presented in Table 1 and Additional file 3: Table S1, respectively. Briefly, in the anti-PD-1 plus chemotherapy group, the median duration of anti-PD-1 therapy was 5 (4–8) cycles. The median follow-up duration was 8.2 months. In the chemotherapy-alone group, the median duration of chemotherapy was 7 (4–10) cycles. The median follow-up duration was 9.4 months.

**Table 1:** Clinical and pathological characteristics of the 63 patients treated with anti-PD-1 plus chemotherapy

Characteristics		Number	Percent
Age, mean (SD)		52.3 (12.7)	
Gender	Male	36	57.1%
	Female	27	42.9%
ECOG PS	0	44	69.8%
	1 ~ 2	19	30.2%
BMI, mean (SD)		21.3 (2.94)	
Tumor location	GEJ	15	23.8%
	Stomach	48	76.2%
Tumor differentiation	Well-moderate	6	9.5%
	Poor	51	81.0%
	Unknown	6	9.5%
Number of organs with metastasis, median (IQR)		2 (1–2)	
Lines of anti-PD-1 therapy	First line	36	57.1%
	Second line or later	27	42.9%
Cycles of anti-PD-1 therapy, median (IQR)		5 (4–8)	
HER2 status	Positive	12	19.0%
	Negative	51	81.0%
PD-L1 expression	CPS ≥ 1	50	79.4%
	CPS < 1	13	20.6%
MMR status	pMMR	53	84.1%
	dMMR	4	6.3%
	Unknown	6	9.5%
EBV status	Positive	4	6.3%
	Negative	51	81.0%
	Unknown	8	12.7%
Disease response (RECIST 1.1)	CR	8	12.7%
	PR	23	36.5%
	SD	18	28.6%
	PD	14	22.2%
NLR, mean (SD)		3.56 (2.71)	
LMR, mean (SD)		3.62 (3.96)	

*ECOG PS* Eastern Cooperative Oncology Group Performance Status, *BMI* Body mass index, *GEJ* gastroesophageal junction, *IQR* interquartile range, *PD-1* programmed death-1, *HER2* human epidermal growth factor receptor-2, *PD-L1* programmed death ligand 1, *CPS* combined positive score, *MMR* mismatch repair, *pMMR* proficient mismatch repair, *dMMR* deficient mismatch repair, *EBV* Epstein-Barr Virus, *CR* complete response, *PR* partial response, *SD* stable disease, *PD* progression disease*, NLR* neutrophil-to-lymphocyte ratio, *LMR* lymphocyte-to-monocyte ratio

As of December 2021, 46 (73.0%) vs. 15 (27.8%) patients were alive and 29 (46.0%) vs. 4 (7.4%) patients were progression-free in the anti-PD-1 plus chemotherapy and chemotherapy-alone groups, respectively. The median PFS and OS were 8.9 (95% CI: 6.9–11.1) vs. 5.37 months (95% CI: 4.0–6.7), not reached vs. 9.37 (95% CI: 7.19–11.5) months in the anti-PD-1 plus chemotherapy and chemotherapy-alone group respectively. As for the tumor response, 8 (12.7%) vs. 0 (0%) patients had complete response (CR), 23 (36.5%) vs. 13 (24.1%) patients had a partial response (PR), 18 (28.6%) vs. 9 (16.7%) patients had stable disease (SD), and 14 (22.2%) vs. 32 (59.3%) patients had a progressive disease (PD) in the anti-PD-1 plus chemotherapy and chemotherapy-alone groups, respectively.

### Higher peripheral basophil counts correlate with non-objective responses to the anti-PD-1 inhibitor plus chemotherapy combination

**Fig. 1:**
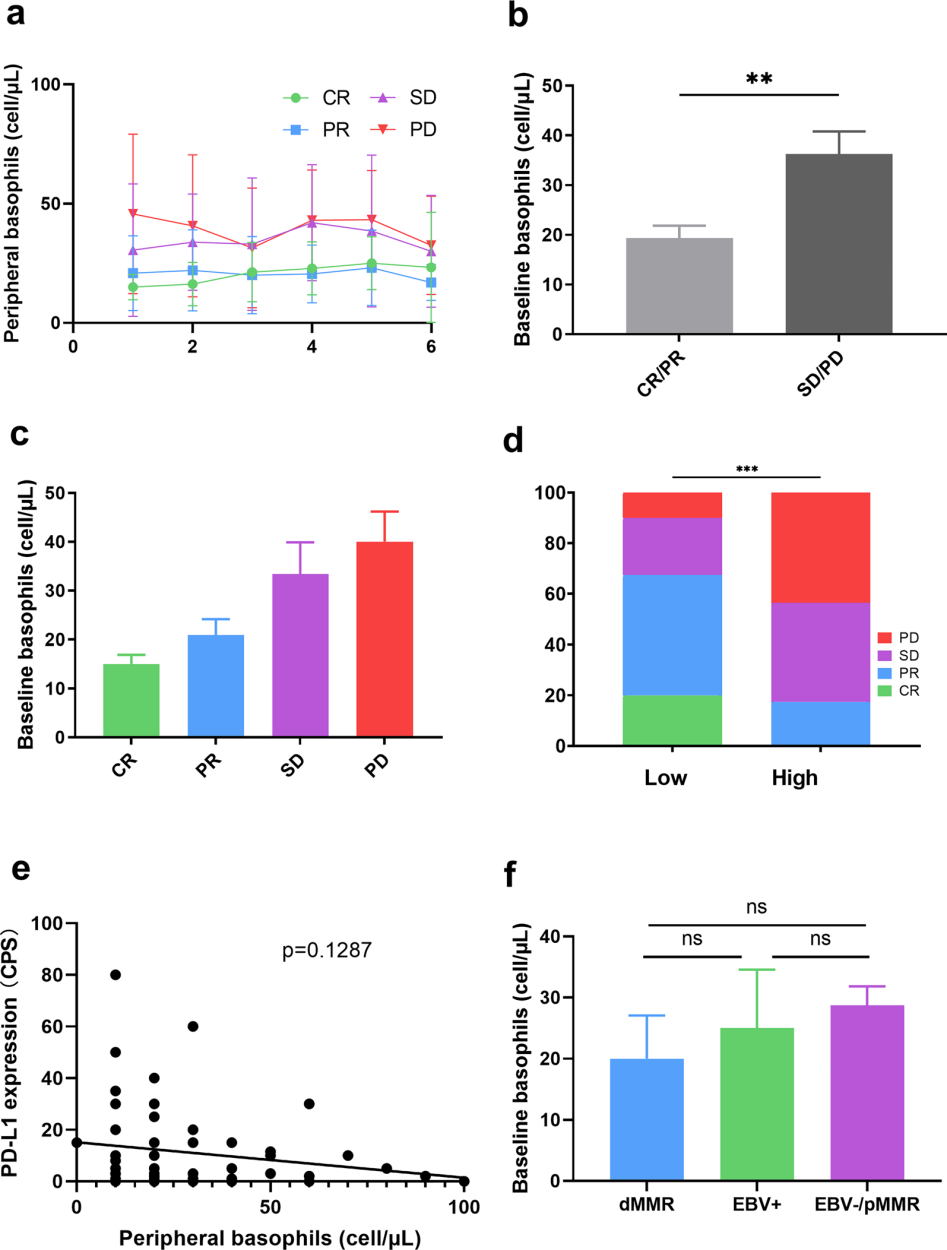
Assessment of peripheral basophils prior to treatment with the anti-PD-1 inhibitor plus chemotherapy combination in patients with advanced gastric cancer. **a** peripheral basophil counts from cycle 1 to cycle 6 of patients according to their disease response. **b** Peripheral basophil counts of patients with gastric cancer who experienced complete/partial response (CR/PR) or stable/progressive disease (SD/PD) as the best objective response to the anti-PD-1 inhibitor plus chemotherapy combination. **c** peripheral basophil counts of patients with CR, PR, SD, or PD. **d** proportions of patients with CR, PR, SD, or PD in the low-basophil and high-basophil groups. **e** Linear correlation between peripheral basophil counts and PD-L1 expression. **f** A comparison of peripheral basophil counts between the dMMR, EBV + , and EBV−/pMMR subgroups. PD-1: programmed death-1; PD-L1: programmed death ligand-1; dMMR: deficient mismatch repair; EBV: Epstein-Barr Virus; pMMR: proficient mismatch repair. Quantitative data are presented as the mean ± SEM. ns indicates not significant, *P < .05, **P < .01

In the anti-PD-1 inhibitor plus chemotherapy group, comparisons of the peripheral leukocyte subpopulation counts at baseline between responders and non-responders revealed differences in the counts of peripheral basophils but not in the counts of neutrophils, eosinophils, monocytes, lymphocytes, the neutrophil-to-lymphocyte ratio (NLR), or the lymphocyte-to-monocyte ratio (LMR) (Additional file 2: Figure S1). In contrast, no significant difference in the peripheral basophil count was noticed between responders and non-responders in the chemotherapy-alone group (Additional file 2: Figure S2). We longitudinally followed the number of peripheral basophils prior to every cycle (Fig. 1a). The mean peripheral basophil counts of CR/PR were always lower than those of SD/PD from cycle 1 to cycle 6. The mean basophil count was 19.35/μL in patients with an objective response, compared with 36.25/μL in patients without an objective response (p = 0.002) (Fig. 1b). At baseline, the mean basophil counts were 40.00/μL cells in patients with PD, 33.33/μL cells in patients with SD, 20.87/μL in patients with PR, and 15.00/μL cells in patients who had CR (Fig. 1c).

An ROC analysis was utilized to assess the optimal peripheral basophil count with respect to OR (Additional file 2: Figure S3a). This value categorized patients into high (> 20.00/μL, n = 23, 36.5%) and low baseline basophil (≤ 20.00/μL, n = 40, 63.5%) groups with an area under the curve of 0.7208 (95% CI: 0.5909–0.8507, p = 0.0026). Baseline clinicopathological characteristics were generally balanced between the two groups in terms of most clinical characteristics except the MMR status (Table 2). Patients with pMMR disease were more common in the low-basophil-count group (p = 0.035). The OR rate for patients with decreased basophil counts was 67.5% (27/40 cases) whereas that for patients with higher basophil counts was 17.4% (4/23 cases; p = 0.0001) (Fig. 1d). The basophil count demonstrated good efficacy in distinguishing responders from non-responders in the EBV-negative, pMMR, first-line, and second-line or later subgroups (Additional file 2: Figure S3b, c, d, e). In contrast, the CPS had no association with treatment response (Additional file 2: Figure S3f).

**Table 2:** Distribution of clinical characteristics by peripheral basophils level

Characteristics		Low basophils (n = 40)		High basophils (n = 23)		p value
Age, mean (SD)		52.0 (14.0)		54.3 (10.0)		0.366
Gender	Male	20	50.0%	16	69.6%	0.131
	Female	20	50.0%	9	30.4%	
ECOG PS	0	27	67.5%	17	73.9%	0.593
	1 ~ 2	13	32.5%	6	26.1%	
BMI, mean (SD)		21.3 (2.91)		21.1 (3.05)		0.687
Tumor location	GEJ	9	22.5%	6	26.1%	0.748
	Stomach	31	77.5%	17	73.9%	
Tumor differentiation	Well-moderate	4	10.0%	2	8.7%	0.968
	Poor	32	80.0%	19	82.6%	
	Unknown	4	10.0%	2	8.7%	
Lines of anti-PD-1 therapy	First line	24	60.0%	12	52.2%	0.546
	Second line or later	16	40.0%	11	47.8%	
Cycles of anti-PD-1 therapy, median (IQR)		5 (4–8)		5 (4–7)		0.549
HER2 status	Positive	5	12.5%	2	8.7%	0.644
	Negative	35	87.5%	21	91.3%	
PD-L1 expression	CPS ≥ 1	33	82.5%	17	73.9%	0.417
	CPS < 1	7	17.5%	6	26.1%	
MMR status	pMMR	36	90.0%	17	73.9%	0.042
	dMMR	3	7.5%	1	4.3%	
	Unknown	1	2.5%	5	21.7%	
EBV status	Positive	2	5.0%	2	8.7%	0.199
	Negative	35	87.5%	16	69.6%	
	Unknown	3	7.5%	5	21.7%	
Number of organs with metastasis, median (IQR)		2.0 (1–2)		2 (1–2)		0.637
NLR, mean (SD)		3.80 (3.15)		2.77 (1.68)		0.357
LMR, mean (SD)		2.91 (4.90)		3.21 (1.16)		0.539

*ECOG PS* Eastern Cooperative Oncology Group Performance Status, *BMI* Body mass index, *GEJ* gastroesophageal junction, *PD-1* programmed death-1, *IQR* interquartile range, *HER2* human epidermal growth factor receptor-2, *PD-L1* programmed death ligand 1, *CPS* combined positive score, *MMR* mismatch repair, *pMMR* proficient mismatch repair, *dMMR* deficient mismatch repair, *EBV* Epstein-Barr Virus, *NLR* neutrophil-to-lymphocyte ratio, *LMR* lymphocyte-to-monocyte ratio

No correlation was detected between peripheral basophil counts and PD-L1 expression (Fig. 1e). No significant differences in basophil counts were noticed between the EBV + , dMMR, or EBV-/pMMR groups (Fig. 1f), supporting the independent association between basophils and responses to the anti-PD-1 inhibitor plus chemotherapy combination. Furthermore, the association between basophils and the treatment response remained statistically significant in the multivariate analysis (adjusted odds ratio = 0.040, 95% CI: 0.007–0.241, p = 0.0004) (Additional file 3: Table S2).

### Higher peripheral basophil counts are prognostic for poor survival with the anti-PD-1 inhibitor plus chemotherapy combination

**Fig. 2:**
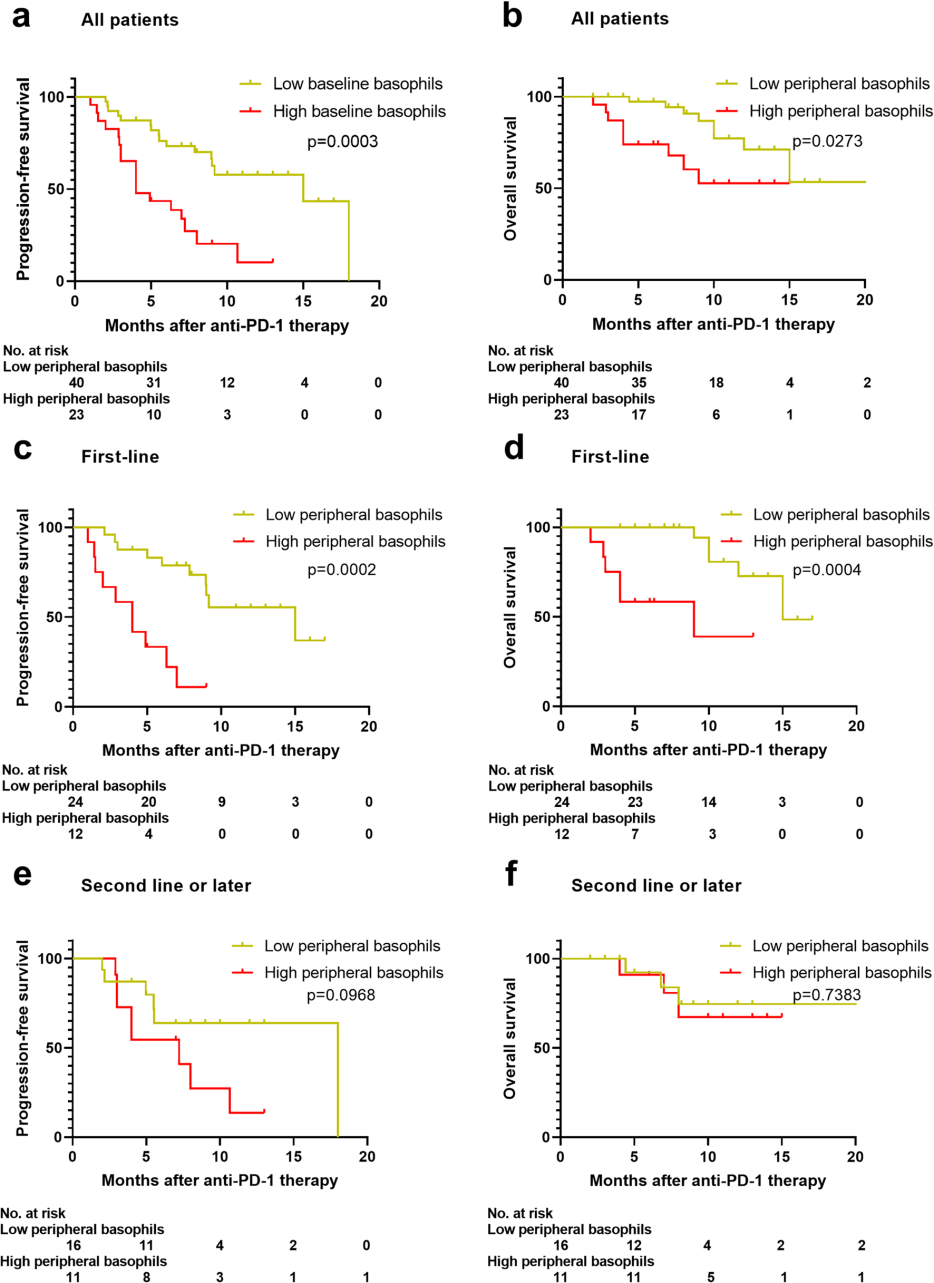
A high peripheral basophil count at baseline was prognostic for unfavorable survival due to the anti-PD-1 plus chemotherapy combination. Progression-free survival and overall survival for all patients (**a**, **b**), patients receiving first-line (**c**, **d**), and second-line or later (**e**, **f**) anti-PD-1 plus chemotherapy stratified by peripheral basophil counts at baseline

For patients treated with the anti-PD-1 inhibitor plus chemotherapy combination, using the absolute basophil count of 20.00/μL as the cut-off, patients with basophil counts of > 20.00/μL had a significantly worse median PFS of 4.0 months (95% CI: 2.2–5.79) than those with basophil counts of ≤ 20.00/μL (median PFS of 15.0 months, 95% CI: 3.73–26.3, p = 0.0001) (Fig. 2a). Patients with baseline basophil counts of > 20.00/μL displayed unfavorable OS (median OS not reached) compared with patients with those with basophil counts of ≤ 20.00/μL (median OS not reached) (p = 0.0273) (Fig. 2b). After adjusting for confounding factors, a high basophil count was identified as an independent adverse prognosticator of PFS [hazard ratio (HR) = 3.720, 95% CI: 1.823–7.594, p = 0.0003] (Additional file 3: Table S3) and OS (HR = 3.427, 95% CI: 1.698–6.917, p = 0.001) (Additional file 3: Table S4). In the chemotherapy-only group, the cutoff value of the basophil count was defined as the mean value or the optimal threshold by selecting the largest χ2 value (most significant association with the OS). We did not observe any survival differences between the two groups stratified by the mean value (p = 0.9135, and p = 0.3117) or the optimal threshold (p = 0.5693, and p = 0.3789) (Additional file 2, Figure S4).

We further stratified the patients by the lines of anti-PD-1 therapy. In the first-line subgroup, higher peripheral basophil counts were still associated with worse PFS and worse OS (p = 0.0002, and p = 0.001) (Fig. 2c, d). However, in the second-line or later subgroup, the basophil count was not associated with OS (p = 0.7383) but tended to correlate with worse PFS though the difference was not statistically significant (p = 0.098) (Fig. 2e, f).

### Early peripheral basophil count changes correlate with clinical outcomes

**Fig. 3:**
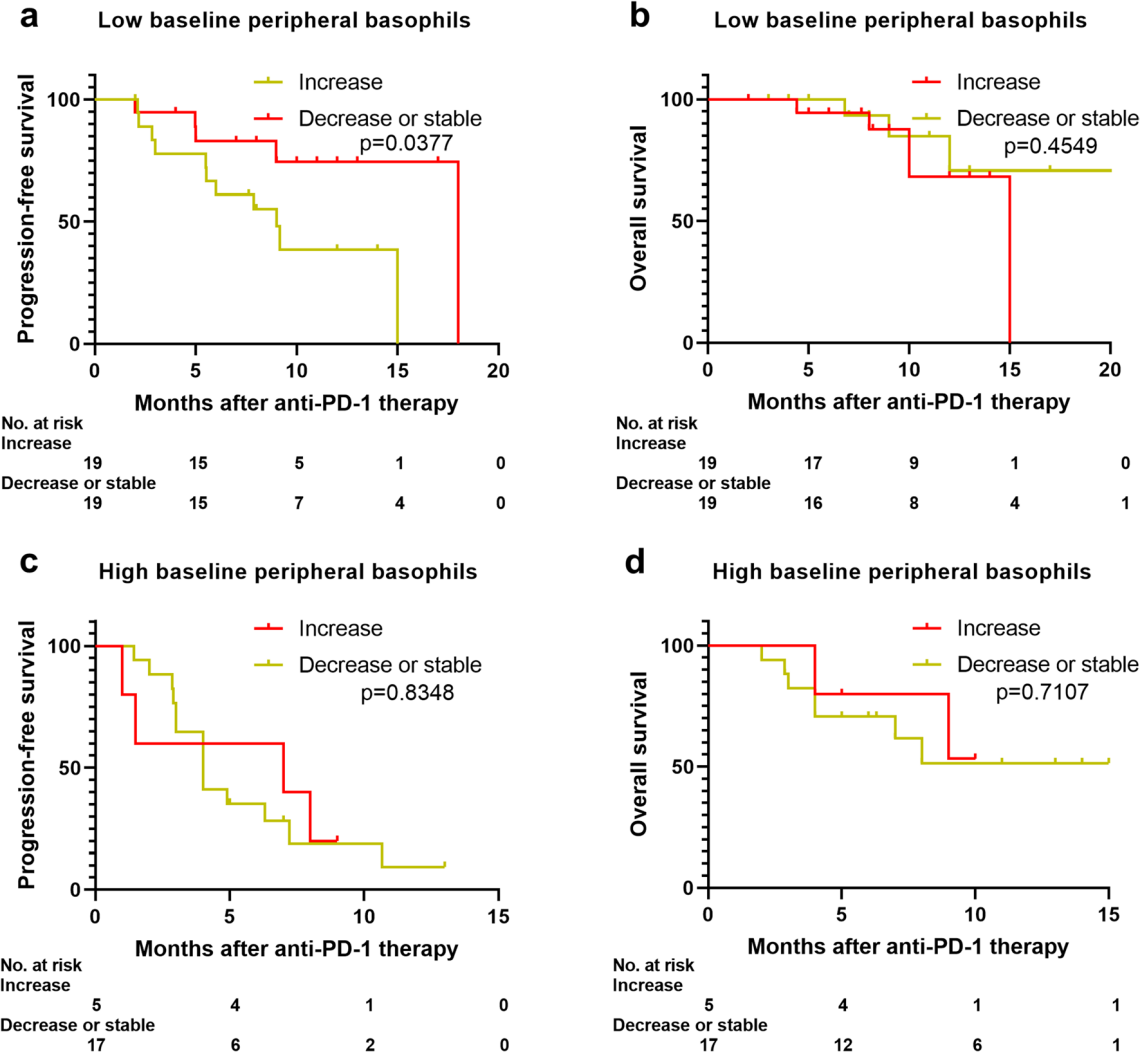
An increase in the peripheral basophil count at cycle 3 correlates with unfavorable survival due to the anti-PD-1 plus chemotherapy combination. **a** PFS and **b** OS of patients with a baseline basophil count of ≤ 20.0/μL grouped as increased or decreased/stable according to the change in the basophil count at cycle 3 of the anti-PD-1 inhibitor plus chemotherapy combination. **c** PFS and **d** OS of patients with a baseline basophil count of > 20.0/μL grouped as increased or decreased/stable according to the change in the basophil count at cycle 3 of the anti-PD-1 inhibitor plus chemotherapy combination

Next, we investigated whether a change in the peripheral basophil count from baseline to cycle 3 of treatment correlates with clinical outcomes of the anti-PD-1 plus chemotherapy combination. Among patients who initially had baseline basophil counts of ≤ 20.0/μL, we found that an increase in the basophil count at cycle 3 was associated with a worse PFS (9.0 months vs. 18.0 months, p = 0.0377) but not OS (15.0 months vs. not reached, p = 0.4549) when compared with patients without increased basophil counts at cycle 3 (Fig. 3a, b). In contrast, among patients with baseline basophil counts of > 20.0/μL, clinical outcomes did not change regardless of subsequent changes in the basophil count at cycle 3 (Fig. 3c, d).

### Association between high basophil counts and increased tumor M2 macrophage infiltration

**Fig. 4:**
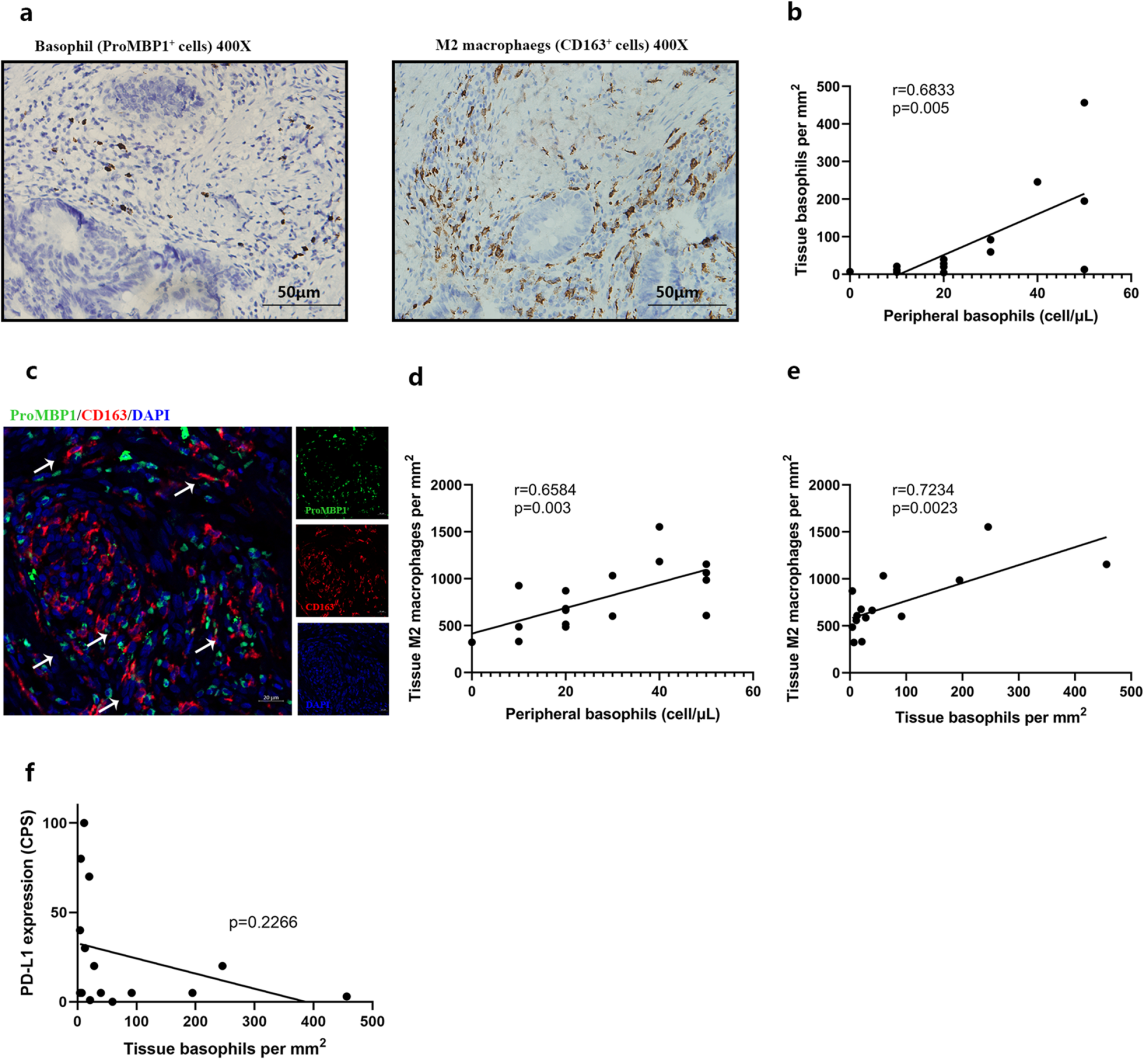
Correlation of tumor infiltration with basophils and M2 macrophages. **a** Representative immunohistochemical images of basophils stained with an anti-ProMBP1 antibody and of M2 macrophages stained with an anti-CD163 antibody on paraffin sections of primary tumors. Scale bar, 50 μm. **b** The peripheral basophil counts were plotted against basophil numbers in primary tumors of corresponding patients. **c** Representative immunofluorescence images of basophils (green) and M2 macrophages (red) in gastric cancer tissues showing their spatial proximity. Scale bar, 20 μm. **d** The peripheral basophil counts were plotted against M2 macrophage counts in primary tumors of corresponding patients. **e**, **f** The tissue basophil counts were plotted against tissue M2 macrophage counts and PD-L1 expression in primary tumors of corresponding patients. *Arrows* show the spatial proximity between basophils and M2 macrophages. ProMBP1 (pro-major basic protein 1), PD-L1, programmed death ligand-1

We first investigated whether peripheral basophil counts were related to intratumoral infiltration. Eighteen treatment-naïve gastric cancer samples were stained with anti-ProMBP1 antibodies against basophils (Fig. 4a). Interestingly, we found a correlation between the peripheral basophil counts prior to surgery and tissue-infiltrating basophil counts of corresponding patients (r = 0.6833, p = 0.005, Fig. 4b). As the most abundant immune cell component in TME, TAMs typically present as M2-like phenotypes, which are well known to induce immunosuppression and resistance to immunotherapy. Since basophils are known to polarize macrophages toward an M2 phenotype [20, 21], we postulated that basophils may orient the TME towards an immune-evasive phenotype by increasing tumor M2 macrophage infiltration. Therefore, treatment-naïve primary tumor samples were stained with antibodies against CD163 (Fig. 4a). Immunofluorescence revealed their spatial proximity within TME (Fig. 4c). It was observed that both the peripheral and tumor-infiltrating basophil counts were positively correlated with the number of tumor-infiltrating M2 macrophages (r = 0.6584, p = 0.003, and r = 0.7234, p = 0.0023) (Fig. 4d, e). The numbers of tumor-infiltrating basophils had no association with PD-L1 expression (Fig. 4f), further suggesting that basophils affected the treatment efficacy independent of PD-L1.

**Fig. 5:**
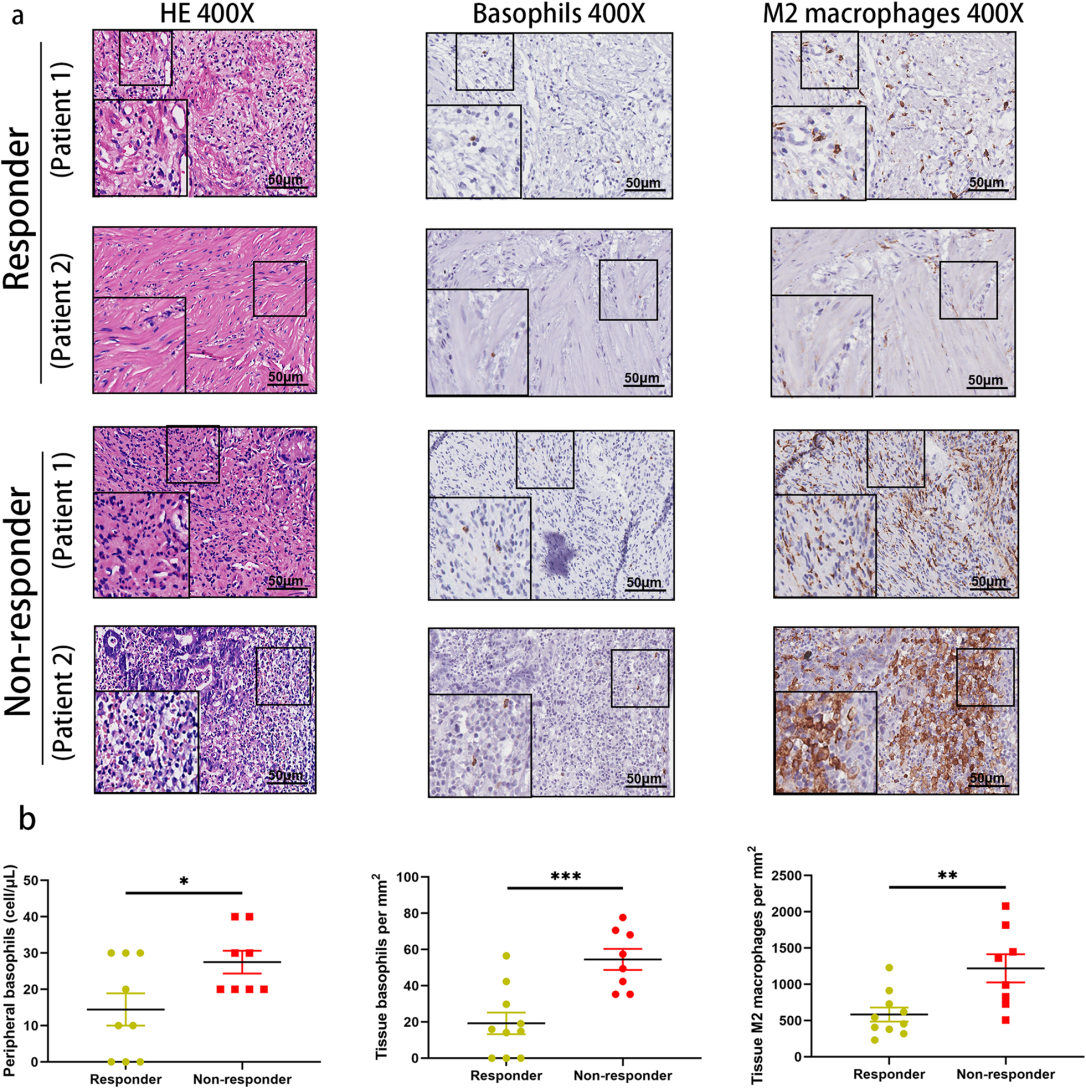
The numbers of peripheral basophils, tumor-infiltrating basophils, and M2 macrophages in patients treated with the neoadjuvant anti-PD-1 inhibitor plus chemotherapy combination. **a** Representative immunohistochemical images of consecutive paraffin sections of primary tumors post-neoadjuvant anti-PD-1 inhibitor plus chemotherapy stained with hematoxylin/eosin (HE), anti-ProMBP1, and anti-CD163 antibodies in responders and non-responders. Scale bar, 50 μm. **b** The peripheral basophils, tumor-infiltrating basophils, and M2 macrophage counts from responders and non-responders. Responders were characterized by tumor regression of grades 0–1. PD-1, programmed death-1. Quantitative data are presented as the mean ± SEM. *P < .05. **P < .01. ***P < .001

Harnessing tumor samples from advanced gastric cancer patients was difficult. To further confirm our conclusion in the context of immunotherapy, we collected primary tumor samples from 18 patients treated with the neoadjuvant anti-PD-1 inhibitor plus chemotherapy combination and performed consecutive staining against basophils and M2 macrophages (Fig. 5a). Consistent with our conclusion, there was a strong trend toward higher counts of peripheral basophils, tissue-infiltrating basophils, and tissue-infiltrating M2 macrophages in the non-responder group than in the responder group (p = 0.0333, p = 0.0007, and p = 0.0066, respectively) (Fig. 5b).

## Discussion

In this study, we reported that among recurrent or metastatic gastric cancer patients treated with the anti-PD-1 inhibitor plus chemotherapy combination, high peripheral basophil counts at baseline and increased basophil counts at cycle 3 compared to baseline were prognostic for unfavorable clinical outcomes. In contrast, no correlation was observed in the chemotherapy-only group. Peripheral basophil counts were positively correlated with the abundance of intratumoral basophils. Both peripheral and intratumoral basophil counts were positively correlated with the abundance of tumor-infiltrating M2 macrophages. Increased basophil counts may indicate an immune-evasive TME characterized by more abundant tumor M2 macrophage infiltration.

Emerging evidence suggests that the efficacy of peripheral cellular biomarkers in improving patient stratification for anti-PD-1 therapy looks promising. Indeed, numerous biomarkers captured in peripheral blood analyses or simple parameters quantified from complete blood routine tests are suggested to be associated with the efficacy of immunotherapy or chemotherapy [22–25]. In this study, decreased peripheral basophil counts at baseline were found to be independently associated with improved radiological responses and was prognostic for favorable survival in advanced gastric cancer. The basophil count might be a potential biomarker of anti-PD-1 efficacy. Consistent with our findings, IL4^+^ basophils were found to reduce the survival of pancreatic cancer patients by regulating Th2 inflammation [26]. Gastric cancer-infiltrating basophils were indicative of worse survival and inferior benefits of adjuvant chemotherapy [27]. In contrast, basophils prolonged the survival of melanoma patients by recruiting CD8^+^ T cells and enhancing tumor rejection [28]. Higher peripheral basophil counts at baseline correlated with longer OS in immune checkpoint inhibitor-treated melanoma [29]. Different tissue microenvironments may account for this discrepancy as different tumor microenvironments may represent distinct cytokine milieus that could modulate the specific gene signature of resident basophils and alter the basophils towards different phenotypes [21]. Although we suggest that peripheral basophil counts were prognostic for survival in the anti-PD-1 plus chemotherapy group but not in the chemotherapy-only group, the definitive role of basophils in immunotherapy is far from clear and warrants further investigation.

Our observation that an early increase in the basophil count from cycle 1 to cycle 3 of anti-PD-1 plus chemotherapy combination in patients with low baseline basophil counts was prognostic for unfavorable clinical outcomes may distinguish patients with a greater probability of disease progression on anti-PD-1 therapy from those with a low risk of progression before radiological assessments. For these patients, peripheral basophil surveillance could potentially guide the implementation of alternative treatment solutions in a timely manner.

Numerous peripheral cellular biomarkers have been demonstrated to predict immune checkpoint inhibitor efficacy; however, few of them have attempted to examine how these biomarkers impact the TME [24, 30]. Basophils are well known to present in the TME and are typically thought to be critical effector cells in allergy and protective immunity after parasite infection [31–33]. In fact, basophils are highly interactive cells. Unique functions unmet by other blood-borne cells, such as immune imprinting, host response to bacteria, autoimmune disease, and allograft fibrosis, have been identified in basophils [21, 34–37]. In contrast to mast cells whose progenitors become fully differentiated in tissues, basophils are thought to complete their development in hematopoietic tissues and keep circulating in the blood until they are cleared or recruited into tissues in pathological conditions [38]. Therefore, we stained the basophils on tumor sections and found that high peripheral basophil counts were associated with increased tumor-infiltrating basophil counts. Although basophil accumulation has been found in tumoral tissues and the peripheral blood of cancer patients [39–43], to our knowledge, this study is the first to show such an association in the same patient. Nevertheless, since basophils had been observed in tumor-draining lymph nodes where they regulated the intratumoral Th2 inflammation [26], it was possible that they may also exert biological effects in other sites in addition to the primary tumor.

Furthermore, it has not been examined if patients who display basophilia before treatment and did not respond to immunotherapy have basophil-mediated immunosuppression. TAMs, a specialized phenotype of M2-polarized macrophages, produced more anti-inflammatory cytokines than M1-type macrophages [11]. These anti-inflammatory cytokines could induce immunosuppression and promote tumor progression and resistance to immunotherapy [44]. We interrogated the basophil and M2 macrophage infiltrates within the TME and found that they were spatially in proximity to each other. Further, we observed that both the peripheral and tumor-infiltrating basophil counts had positive correlations with the tumor-infiltrating M2 macrophage count that characterized an immune-evasive TME. Consistent with our findings, tumor-infiltrating basophils had been demonstrated to be associated with M2-polarized macrophage infiltration and decreased interferon-γ expression in gastric cancer [27]. It has also been shown that basophils regulate alveolar macrophage maturation and immunomodulation function [21]. In line with this observation, our results give some indications that basophils might modulate the local TME and contribute to immune suppression. To further confirm this conclusion in the context of anti-PD-1 plus chemotherapy, primary tumor samples from gastric cancer patients treated with the neoadjuvant anti-PD-1 plus chemotherapy combination were collected and stained against proMBP1 and CD163. Consistent with our conclusion, the responders have decreased peripheral basophil counts and tumor infiltration of basophils and M2 macrophages.

This study has several limitations that could not be neglected. First, its retrospective design has the inherent deficit of being observational or non-experimental. For example, variables such as allergic conditions or medications that potentially affect the circulating basophil count were not fully considered. Second, only M2 macrophages within the TME were investigated in this study. Basophils may have effects on other immune cell subpopulations in addition to M2 macrophages since they could broadly interact with the immune and non-immune compartmentsyy [21]. The whole immune landscape of gastric cancer needs to be further investigated in further studies. Lastly, the optimal basophil count threshold needs to be prospectively validated in a multicenter randomized controlled trial.

In conclusion, advanced gastric cancer with high peripheral basophil counts at baseline has an M2 macrophage-infiltrating TME and unfavorable clinical outcomes of the anti-PD-1 inhibitor plus chemotherapy combination. Basophil counts may be associated with an immune-evasive tumor microenvironment via an increase in M2 macrophage infiltration, and this could be a potential biomarker of anti-PD-1 efficacy and an immunotherapeutic target for advanced gastric cancer.

## Supplementary Information

**Additional file 1. **Additional methods.

**Additional file 2: Figure S1. **Counts of various peripheral leukocyte populations before treatment with the anti-PD-1 inhibitor plus chemotherapy combination. Peripheral neutrophils (a), monocytes (b), eosinophils (c), lymphocytes (d), neutrophil-lymphocyte ratio (e), and the lymphocyte-to-monocyte ratio (f) from patients with gastric cancer who experienced complete/partial response (CR/PR) or stable/progressive disease (SD/PD) as the best objective response to the anti-PD-1 inhibitor plus chemotherapy combination. **Figure S2.** Peripheral basophils of patients treated with chemotherapy alone who experienced complete/partial response (CR/PR) or stable/progressive disease (SD/PD) as the best objective response to chemotherapy. **Figure S3.** The efficacy of peripheral basophil counts or the CPS in distinguishing responders from non-responders to the anti-PD-1 inhibitor plus chemotherapy combination. Receiver operating characteristic (ROC) curve to evaluate the performance of peripheral basophils at baseline for identifying patients with a response (CR/PR) in (a) all patients, (b) EBV-negative, (c) pMMR, (d) first-line, (e) second-line or later subgroups. (f) ROC curve to evaluate the performance of CPS for identifying patients with a response. **Figure S4. **The peripheral basophil count at baseline was not prognostic for survival due to chemotherapy alone. The progression-free survival and overall survival of patients treated with chemotherapy alone stratified by the mean value (a, b) or the optimal cut-off value (c, d) of the peripheral basophil count at baseline.

**Additional file 3: Table S1.** Clinical and pathological characteristics of the 54 patients treated with chemotherapy alone. **Table S2.** Univariate and multivariate logistic regression analyses of objective response in patients with advanced gastric cancer treated with the anti-PD-1 inhibitor plus chemotherapy combination. **Table S3.** Univariate and multivariate Cox regression analyses of progression-free survival in patients with advanced gastric cancer treated with the anti-PD-1 inhibitor plus chemotherapy combination. **Table S4. **Univariate and multivariate Cox regression analyses of overall survival in patients with advanced gastric cancer treated with the anti-PD-1 inhibitor plus chemotherapy combination.

## Data Availability

Data are available upon reasonable request.

## Abbreviations

PD-1: Anti-programmed death-1
ORR: Objective response rate
PFS: Progression-free survival
OS: Overall survival
CI: Confidence interval
ICI: Immune checkpoint inhibitor
MSI: Microsatellite instability
MMR: Mismatch repair
EBV: Epstein-Barr virus
TMB: Tumor mutation burden
TME: Tumor microenvironment
TAM: Tumor-associated macrophage
IF: Immunofluorescence
IHC: Immunohistochemistry
CR: Complete response
PR: Partial response
SD: Stable disease
PD: Progressive disease
HER2: Human epidermal growth factor receptor 2
EBER: EBV-encoded small RNA
ProMBP1: Anti-Pro Major Basic Protein 1
ROC: Receiver operating characteristic
IQR: Interquartile range
dMMR: Deficient mismatch repair
pMMR: Proficient mismatch repair
PD-L1: Programmed death ligand-1
CPS: Combined positive score
NLR: Neutrophil-to-lymphocyte ratio
LMR: Lymphocyte-to-monocyte ratio
