# Supplementary material for: Association of peripheral basophils with tumor M2 macrophage infiltration and outcomes of the anti-PD-1 inhibitor plus chemotherapy combination in advanced gastric cancer
Source: J Transl Med. 2022 Sep 4;20:386. doi: 10.1186/s12967-022-03598-y (PMC9441040; doi:10.1186/s12967-022-03598-y)
Supplement: Supplementary file 1 — Additional file 1. Additional methods. [file 12967_2022_3598_MOESM1_ESM.docx]

Supplementary methods for

Association of peripheral basophils with tumor M2 macrophage infiltration and outcomes of the anti-PD-1 inhibitor plus chemotherapy combination in advanced gastric cancer

**Hematoxylin-eosin staining, immunofluorescence, and immunohistochemistry**

Tumor samples were collected from 18 gastric cancer patients who did not receive any preoperative therapy as well as 18 patients receiving neoadjuvant anti-PD-1 inhibitor plus chemotherapy and curative surgery. Immunohistochemical staining was conducted on 4-µm sections of formalin-fixed paraffin-embedded tumor tissues. In brief, the slides were baked at 60 ℃ for 3 h, deparaffinized in xylene (three times, 15 min each, at room temperature) and rehydrated in graded alcohol. Endogenous peroxidase was blocked with 3% H_2_O_2_ in methanol at 37 ℃ for 30 min. Next, the slides were immersed in 0.01 M citrate buffer (pH 6.0), cooked for antigen retrieval, and then incubated with 10% normal goat serum at 37 ℃ to reduce nonspecific reactions. Subsequently, the slides were incubated with anti-Pro Major Basic Protein 1 (ProMBP1) antibody (Biolegend, catalogue number: 346802, dilution: 1:100) against basophils and anti-CD163 antibody (Abcam, catalogue number: ab182422, dilution: 1:500) against M2 macrophages overnight at 4 ℃. After rinsing three times with 0.01 M phosphate buffer (pH 8.0), for IHC, the slides were incubated with secondary antibody for 20 min at 37 ℃ and stained using diaminobenzidine (DAB)-H_2_O_2_. Ultimately, the slides were counterstained with hematoxylin, dehydrated, and mounted with a coverslip and neutral resins. For immunofluorescence staining, dylight-488 and dylight-649 fluorescence-labelled secondary antibodies were applied and DAPI was counterstained for 5 min at room temperature in the dark. Slides were visualized and imaged using a confocal microscope (LSM980, Zeiss, Germany) with a 10x objective. Simultaneously, a negative control without the first antibody and a verified positive control were stained to avoid false positive or negative results.

**Analysis of stained tumor samples**

Each tumor sample was sectioned into an intratumoral region and a surrounding peritumoral region. At low power (100x), the tissue sections were screened using an upright research microscope (BX51, Olympus, Japan), and the three most representative fields were selected. The mean number of basophils and CD163+macrophages was determined as the mean number of these cells in three randomly selected high-power fields (400x). The average density was defined as the observed cell numbers divided by the evaluated area. Two pathologists who were blinded to clinical characteristics separately counted the cells. Their results were completely consistent with each other in roughly 87% of the cases. A third pathologist was consulted when discrepancies arose between the two primary pathologists. If the third pathologist agreed with one of them, then that value was selected. If the third pathologist had a different result, then all of them worked together to reach a consensus.
