# Supplementary material for: Association of peripheral basophils with tumor M2 macrophage infiltration and outcomes of the anti-PD-1 inhibitor plus chemotherapy combination in advanced gastric cancer
Source: J Transl Med. 2022 Sep 4;20:386. doi: 10.1186/s12967-022-03598-y (PMC9441040; doi:10.1186/s12967-022-03598-y)
Supplement: Supplementary file 2 — Additional file 2: Figure S1. Counts of various peripheral leukocyte populations before treatment with the anti-PD-1 inhibitor plus chemotherapy combination. Peripheral neutrophils (a), monocytes (b), eosinophils (c), lymphocytes (d), neutrophil-lymphocyte ratio (e), and the lymphocyte-to-monocyte ratio (f) from patients with gastric cancer who experienced complete/partial response (CR/PR) or stable/progressive disease (SD/PD) as the best objective response to the anti-PD-1 inhibitor plus chemotherapy combination. Figure S2. Peripheral basophils of patients treated with chemotherapy alone who experienced complete/partial response (CR/PR) or stable/progressive disease (SD/PD) as the best objective response to chemotherapy. Figure S3. The efficacy of peripheral basophil counts or the CPS in distinguishing responders from non-responders to the anti-PD-1 inhibitor plus chemotherapy combination. Receiver operating characteristic (ROC) curve to evaluate the performance of peripheral basophils at baseline for identifying patients with a response (CR/PR) in (a) all patients, (b) EBV-negative, (c) pMMR, (d) first-line, (e) second-line or later subgroups. (f) ROC curve to evaluate the performance of CPS for identifying patients with a response. Figure S4. The peripheral basophil count at baseline was not prognostic for survival due to chemotherapy alone. The progression-free survival and overall survival of patients treated with chemotherapy alone stratified by the mean value (a, b) or the optimal cut-off value (c, d) of the peripheral basophil count at baseline. [file 12967_2022_3598_MOESM2_ESM.docx]

Supplementary figures for

Association of peripheral basophils with tumor M2 macrophage infiltration and outcomes of the anti-PD-1 inhibitor plus chemotherapy combination in advanced gastric cancer

**This file includes:**

**Figure S1.** Counts of various peripheral leukocyte populations before treatment with the anti-PD-1 inhibitor plus chemotherapy combination.

**Figure S2.** Peripheral basophils of patients treated with chemotherapy alone who experienced complete/partial response (CR/PR) or stable/progressive disease (SD/PD) as the best objective response to chemotherapy.

**Figure S3.** The efficacy of peripheral basophil counts or the CPS in distinguishing responders from non-responders to the anti-PD-1 inhibitor plus chemotherapy combination.

**Figure S4.** The peripheral basophil count at baseline was not prognostic for survival due to chemotherapy alone.


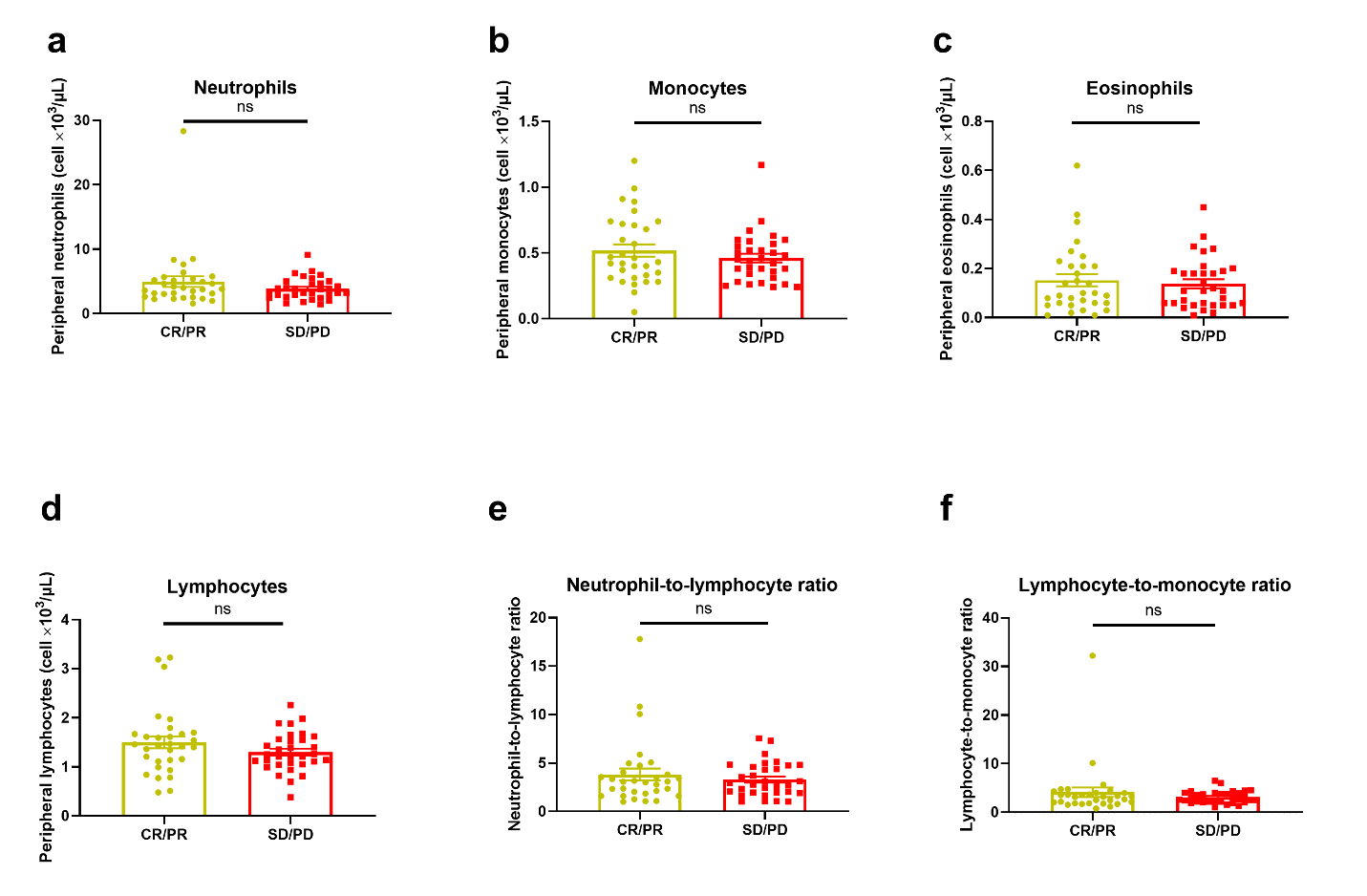


**Figure S1.** Counts of various peripheral leukocyte populations before treatment with the anti-PD-1 inhibitor plus chemotherapy combination. Peripheral neutrophils (a), monocytes (b), eosinophils (c), lymphocytes (d), neutrophil-lymphocyte ratio (e), and the lymphocyte-to-monocyte ratio (f) from patients with gastric cancer who experienced complete/partial response (CR/PR) or stable/progressive disease (SD/PD) as the best objective response to the anti-PD-1 inhibitor plus chemotherapy combination.
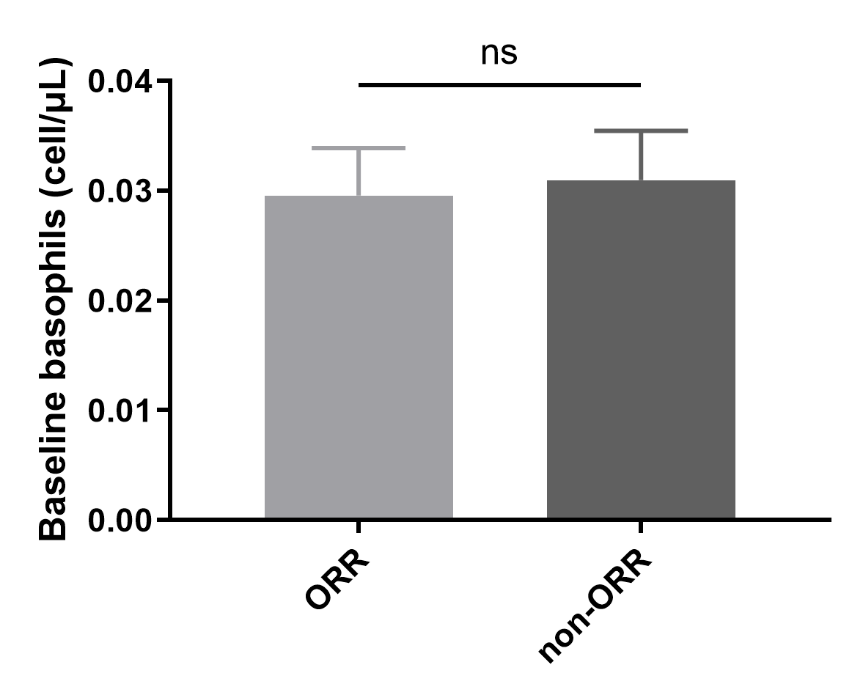


**Figure S2.** Peripheral basophils of patients treated with chemotherapy alone who experienced complete/partial response (CR/PR) or stable/progressive disease (SD/PD) as the best objective response to chemotherapy.


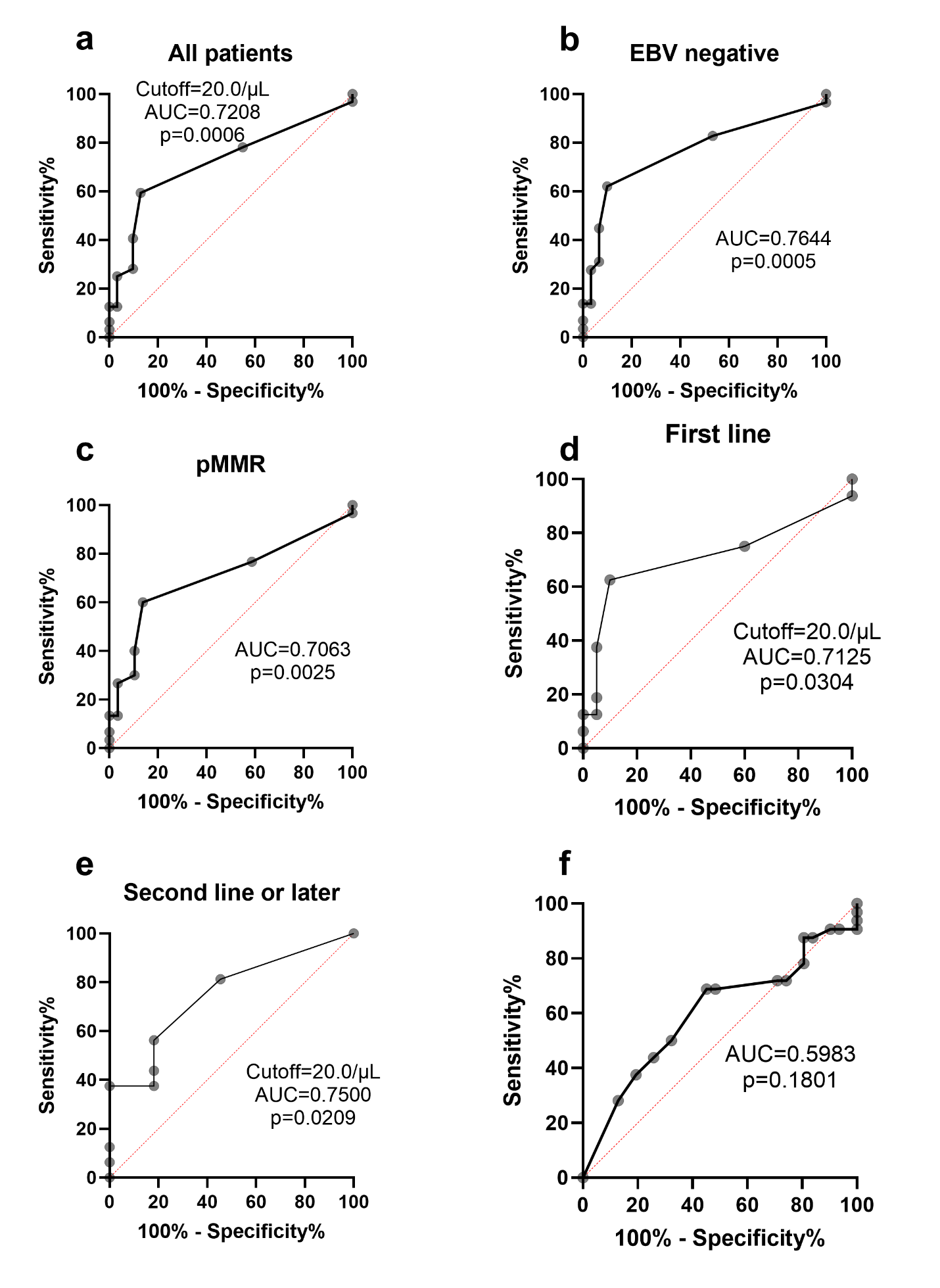


**Figure S3.** The efficacy of peripheral basophil counts or the CPS in distinguishing responders from non-responders to the anti-PD-1 inhibitor plus chemotherapy combination. Receiver operating characteristic (ROC) curve to evaluate the performance of peripheral basophils at baseline for identifying patients with a response (CR/PR) in (a) all patients, (b) EBV-negative, (c) pMMR, (d) first-line, (e) second-line or later subgroups. (f) ROC curve to evaluate the performance of CPS for identifying patients with a response.


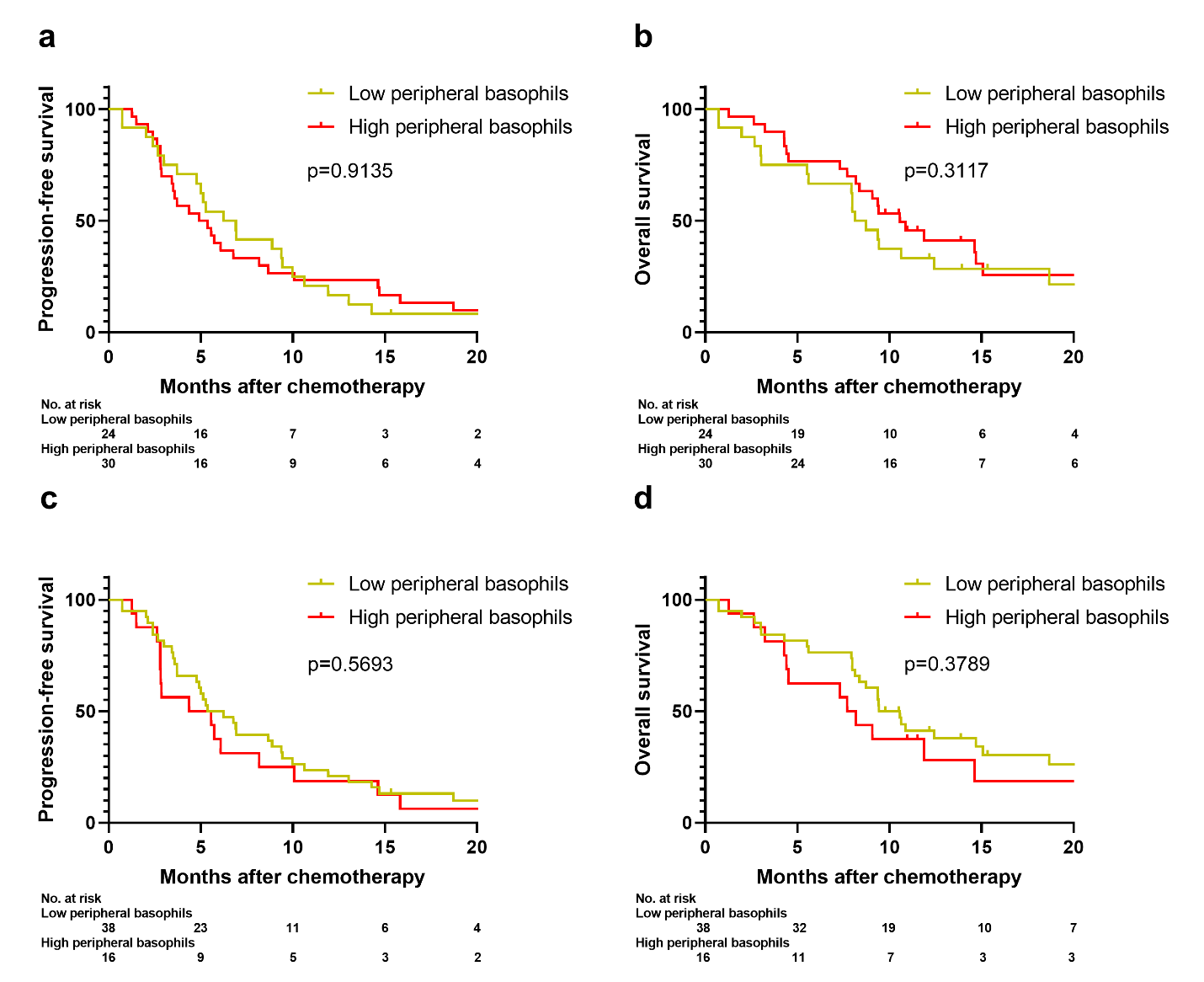


**Figure S4.** The peripheral basophil count at baseline was not prognostic for survival due to chemotherapy alone. The progression-free survival and overall survival of patients treated with chemotherapy alone stratified by the mean value (a, b) or the optimal cut-off value (c, d) of the peripheral basophil count at baseline.
