# Supplementary material for: Association of peripheral basophils with tumor M2 macrophage infiltration and outcomes of the anti-PD-1 inhibitor plus chemotherapy combination in advanced gastric cancer
Source: J Transl Med. 2022 Sep 4;20:386. doi: 10.1186/s12967-022-03598-y (PMC9441040; doi:10.1186/s12967-022-03598-y)
Supplement: Supplementary file 3 — Additional file 3: Table S1. Clinical and pathological characteristics of the 54 patients treated with chemotherapy alone. Table S2. Univariate and multivariate logistic regression analyses of objective response in patients with advanced gastric cancer treated with the anti-PD-1 inhibitor plus chemotherapy combination. Table S3. Univariate and multivariate Cox regression analyses of progression-free survival in patients with advanced gastric cancer treated with the anti-PD-1 inhibitor plus chemotherapy combination. Table S4. Univariate and multivariate Cox regression analyses of overall survival in patients with advanced gastric cancer treated with the anti-PD-1 inhibitor plus chemotherapy combination. [file 12967_2022_3598_MOESM3_ESM.docx]

Supplementary tables for

**Association of peripheral basophils with tumor M2 macrophage infiltration and outcomes of the anti-PD-1 inhibitor plus chemotherapy combination in advanced gastric cancer**

**This file includes:**

**Table S1.** Clinical and pathological characteristics of the 54 patients treated with chemotherapy alone.

**Table S2.** Univariate and multivariate logistic regression analyses of objective response in patients with advanced gastric cancer treated with the anti-PD-1 inhibitor plus chemotherapy combination.

**Table S3.** Univariate and multivariate Cox regression analyses of progression-free survival in patients with advanced gastric cancer treated with the anti-PD-1 inhibitor plus chemotherapy combination.

**Table S4.** Univariate and multivariate Cox regression analyses of overall survival in patients with advanced gastric cancer treated with the anti-PD-1 inhibitor plus chemotherapy combination.

**Table S1.** Clinical and pathological characteristics of the 54 patients treated with chemotherapy alone.

| **Characteristics** |  | **Number** | **Percent** |
| --- | --- | --- | --- |
| **Age, mean (SD)** | 57.0±13.0 |  |  |
| **Gender** | Male | 35 | 64.8% |
|  | Female | 19 | 35.2% |
| **ECOG PS^[[1]](#footnote-1)^** | 0 | 30 | 55.6% |
|  | 1~2 | 24 | 44.4% |
| **BMI^[[2]](#footnote-2)^, mean (SD)** | 20.0 (2.97) |  |  |
| **Tumor location** | GEJ^[[3]](#footnote-3)^ | 15 | 27.8% |
|  | Stomach | 39 | 72.2% |
| **Tumor differentiation** | Well-moderate | 11 | 20.4% |
|  | Poor | 35 | 64.8% |
|  | Unknown | 8 | 14.8% |
| **Number of organs with metastasis, median (IQR)** | 1(1-2) |  |  |
| **Cycles of chemotherapy, median (IQR)** | 7 (4-10) |  |  |
| **HER2^[[4]](#footnote-4)^ status** | Positive | 2 | 13.7% |
|  | Negative | 33 | 71.1% |
|  | Unknown | 19 | 15.2% |
| **Disease response (RECIST 1.1)** | CR^[[5]](#footnote-5)^ | 0 | 0% |
|  | PR^[[6]](#footnote-6)^ | 13 | 24.1% |
|  | SD^[[7]](#footnote-7)^ | 9 | 16.7% |
|  | PD^[[8]](#footnote-8)^ | 32 | 59.3% |

**Table S2.** Univariate and multivariate logistic regression analyses of objective response in patients with advanced gastric cancer treated with the anti-PD-1 inhibitor plus chemotherapy combination.

| **Objective response** | **Univariate odds ratio (95% CI^[[9]](#footnote-9)^)** | **p-value** | **Multivariate odds ratio (95% CI)** | **p-value** |
| --- | --- | --- | --- | --- |
| **Age (＜60 vs. ≥ 60)** | 1.429 (0.476-4.286) | 0.525 |  |  |
| **Gender (male vs. female)** | 1.204 (0.443-3.269) | 0.716 |  |  |
| **ECOG PS^[[10]](#footnote-10)^ (0 vs. 1-2)** | 1.650 (0.556-4.893) | 0.367 |  |  |
| **BMI^[[11]](#footnote-11)^ (≥24 vs.＜24)** | 3.955 (0.957-16.349) | 0.058 | 10.534 (1.078-102.8) | 0.043 |
| **Tumor differentiation (poor/undifferentiated vs. well/mod)** | 1.203 (0.545-2.656) | 0.648 |  |  |
| **Location (GEJ^[[12]](#footnote-12)^ vs. stomach)** | 1.143 (0.358-3.651) | 0.822 |  |  |
| **Number of organs with metastasis (＞2 vs. ≤2)** | 1.083 (0.395-2.974) | 0.877 |  |  |
| **Lines of anti-PD-1^[[13]](#footnote-13)^ therapy (first line vs. second line or later)** | 0.550 (0.200-1.511) | 0.246 |  |  |
| **Cycles of anti-PD-1 therapy (≥5 vs.＜5)** | 2.643 (0.953-7.333) | 0.062 | 4.569 (1.150-18.152) | 0.031 |
| **PD-L1^[[14]](#footnote-14)^ expression (CPS^[[15]](#footnote-15)^≥1 vs. ＜1)** | 2.641 (0.718-9.716) | 0.144 |  |  |
| **HER2^[[16]](#footnote-16)^ status (Pos. vs. neg)** | 7.440 (0.840-65.920) | 0.071 | 6.702 (0.543-82.732) | 0.138 |
| **MMR^[[17]](#footnote-17)^ status (dMMR^[[18]](#footnote-18)^ vs. pMMR^[[19]](#footnote-19)^)** | 1.034 (0.136-7.840) | 0.974 |  |  |
| **EBV^[[20]](#footnote-20)^ status (Pos. vs. neg)** | 0.339 (0.032-3.279) | 0.339 |  |  |
| **NLR^[[21]](#footnote-21)^ (high vs. low)** | 1.100 (0.386-3.139) | 0.859 |  |  |
| **LMR^[[22]](#footnote-22)^ (high vs. low)** | 1.214 (0.451-3.269) | 0.701 |  |  |
| **Baseline peripheral basophils (high vs. low)** | 0.101 (0.029-0.359) | 0.0003 | 0.040 (0.007-0.241) | 0.0004 |

**Table S3.** Univariate and multivariate Cox regression analyses of progression-free survival in patients with advanced gastric cancer treated with the anti-PD-1 inhibitor plus chemotherapy combination.

| **Progression-free survival** | **Univariate hazard ratio (95% CI^[[23]](#footnote-23)^)** | **p-value** | **Multivariate hazard ratio (95% CI)** | **p-value** |
| --- | --- | --- | --- | --- |
| **Age (＜60 vs. ≥60)** | 1.267 (0.614-2.617) | 0.522 |  |  |
| **Gender (male vs. female)** | 0.730 (0.354-1.508) | 0.730 |  |  |
| **ECOG PS^[[24]](#footnote-24)^ (0 vs. 1-2)** | 1.364 (0.670-2.777) | 0.392 |  |  |
| **BMI^[[25]](#footnote-25)^ (≥24 vs.＜24)** | 0.350 (0.106-1.156) | 0.085 | 0.332 (0.100-1.096) | 0.070 |
| **Tumor differentiation (poor/undifferentiated vs. well/mod)** | 0.752 (0.415-1.361) | 0.346 |  |  |
| **Location (GEJ^[[26]](#footnote-26)^ vs. stomach)** | 1.114 (0.481-2.578) | 0.801 |  |  |
| **Number of organs with metastasis (＞2 vs. ≤2)** | 1.662 (0.766-3.602) | 0.198 |  |  |
| **Lines of anti-PD-1^[[27]](#footnote-27)^ therapy (first line vs. second line or later)** | 0.914 (0.454-1.841) | 0.801 |  |  |
| **Cycles of anti-PD-1 therapy (≥5 vs.＜5)** | 0.296 (0.340-1.390) | 0.296 |  |  |
| **PD-L1^[[28]](#footnote-28)^ expression (CPS^[[29]](#footnote-29)^≥1 vs. ＜1)** | 1.700 (0.653-4.426) | 0.277 |  |  |
| **HER2^[[30]](#footnote-30)^ status (Pos. vs. neg)** | 0.218 (0.030-1.599) | 0.134 |  |  |
| **MMR^[[31]](#footnote-31)^ status (dMMR^[[32]](#footnote-32)^ vs. pMMR^[[33]](#footnote-33)^)** | 1.590 (0.374-6.760) | 0.530 |  |  |
| **EBV^[[34]](#footnote-34)^ status (Pos. vs. neg)** | 2.379 (0.818-6.918) | 0.112 |  |  |
| **NLR^[[35]](#footnote-35)^ (high vs. low)** | 1.297 (0.616-2.729) | 0.494 |  |  |
| **LMR^[[36]](#footnote-36)^ (high vs. low)** | 0.620 (0.312-1.233) | 0.173 |  |  |
| **Baseline peripheral basophils (high vs. low)** | 3.629 (1.788-7.407) | 0.0003 | 3.720 (1.823-7.594) | 0.0003 |

**Table S4.** Univariate and multivariate Cox regression analyses of overall survival in patients with advanced gastric cancer treated with the anti-PD-1 inhibitor plus chemotherapy combination.

| **Overall survival** | **Univariate hazard ratio (95% CI^[[37]](#footnote-37)^)** | **p-value** | **Multivariate hazard ratio (95% CI)** | **p-value** |
| --- | --- | --- | --- | --- |
| **Age (＜60 vs. ≥60)** | 1.024 (0.495-2.117) | 0.949 |  |  |
| **Gender (male vs. female)** | 0.959 (0.463-1.986) | 0.910 |  |  |
| **ECOG PS^[[38]](#footnote-38)^ (0 vs. 1-2)** | 1.115 (0.547-2.270) | 0.765 |  |  |
| **BMI^[[39]](#footnote-39)^ (≥24 vs.＜24)** | 0.395 (0.119-1.310) | 0.129 |  |  |
| **Tumor differentiation (poor/undifferentiated vs. well/mod)** | 0.517 (0.461-1.475) | 0.517 |  |  |
| **Location (GEJ^[[40]](#footnote-40)^ vs. stomach)** | 1.124 (0.483-2.615) | 0.787 |  |  |
| **Number of organs with metastasis (＞2 vs ≤2)** | 1.295 (0.588-2.852) | 0.521 |  |  |
| **Lines of anti-PD-1^[[41]](#footnote-41)^ therapy (first line vs. second line or later)** | 0.952 (0.473-1.917) | 0.891 |  |  |
| **Cycles of anti-PD-1 therapy (≥ 5 vs.＜5)** | 0.518 (0.248-1.083) | 0.081 | 0.424 (0.197-0.908) | 0.027 |
| **PD-L1^[[42]](#footnote-42)^ expression (CPS^[[43]](#footnote-43)^≥1 vs. ＜1)** | 1.434 (0.548-3.750) | 0.463 |  |  |
| **HER2^[[44]](#footnote-44)^ status (Pos. vs. neg)** | 0.281 (0.038-3.065) | 0.212 |  |  |
| **MMR^[[45]](#footnote-45)^ status (dMMR^[[46]](#footnote-46)^ vs. pMMR^[[47]](#footnote-47)^)** | 2.170 (0.493-9.551) | 0.306 |  |  |
| **EBV^[[48]](#footnote-48)^ status (Pos. vs. neg)** | 2.225 (0.761-6.501) | 0.144 |  |  |
| **NLR^[[49]](#footnote-49)^ (high vs. low)** | 0.877 (0.331-2.325) | 0.792 |  |  |
| **LMR^[[50]](#footnote-50)^ (high vs. low)** | 0.667 (0.255-1.745) | 0.409 |  |  |
| **Baseline peripheral basophils (high vs. low)** | 3.018 (1.514-6.015) | 0.002 | 3.427 (1.698-6.917) | 0.001 |

1. Eastern Cooperative Oncology Group Performance Status [↑](#footnote-ref-1)
2. Body mass index [↑](#footnote-ref-2)
3. Gastroesophageal junction [↑](#footnote-ref-3)
4. Human epidermal growth factor receptor-2 [↑](#footnote-ref-4)
5. Complete response [↑](#footnote-ref-5)
6. Partial response [↑](#footnote-ref-6)
7. Stable disease [↑](#footnote-ref-7)
8. Progression disease [↑](#footnote-ref-8)
9. Confidence interval [↑](#footnote-ref-9)
10. Eastern Cooperative Oncology Group Performance Status [↑](#footnote-ref-10)
11. Body mass index [↑](#footnote-ref-11)
12. Gastroesophageal junction [↑](#footnote-ref-12)
13. Programmed death 1 [↑](#footnote-ref-13)
14. Programmed death ligand 1 [↑](#footnote-ref-14)
15. Combined positive score [↑](#footnote-ref-15)
16. Human epidermal growth factor receptor-2 [↑](#footnote-ref-16)
17. Mismatch repair [↑](#footnote-ref-17)
18. Deficient mismatch repair [↑](#footnote-ref-18)
19. Proficient mismatch repair [↑](#footnote-ref-19)
20. Epstein-Barr Virus [↑](#footnote-ref-20)
21. Neutrophil-to-lymphocyte ratio [↑](#footnote-ref-21)
22. Lymphocyte-to-monocyte ratio [↑](#footnote-ref-22)
23. Confidence interval [↑](#footnote-ref-23)
24. Eastern Cooperative Oncology Group Performance Status [↑](#footnote-ref-24)
25. Body mass index [↑](#footnote-ref-25)
26. Gastroesophageal junction [↑](#footnote-ref-26)
27. Programmed death 1 [↑](#footnote-ref-27)
28. Programmed death ligand 1 [↑](#footnote-ref-28)
29. Combined positive score [↑](#footnote-ref-29)
30. Human epidermal growth factor receptor-2 [↑](#footnote-ref-30)
31. Mismatch repair [↑](#footnote-ref-31)
32. Deficient mismatch repair [↑](#footnote-ref-32)
33. Proficient mismatch repair [↑](#footnote-ref-33)
34. Epstein-Barr Virus [↑](#footnote-ref-34)
35. Neutrophil-to-lymphocyte ratio [↑](#footnote-ref-35)
36. Lymphocyte-to-monocyte ratio [↑](#footnote-ref-36)
37. Confidence interval [↑](#footnote-ref-37)
38. Eastern Cooperative Oncology Group Performance Status [↑](#footnote-ref-38)
39. Body mass index [↑](#footnote-ref-39)
40. Gastroesophageal junction [↑](#footnote-ref-40)
41. Programmed death-1 [↑](#footnote-ref-41)
42. Programmed death ligand 1 [↑](#footnote-ref-42)
43. Combined positive score [↑](#footnote-ref-43)
44. Human epidermal growth factor receptor-2 [↑](#footnote-ref-44)
45. Mismatch repair [↑](#footnote-ref-45)
46. Deficient mismatch repair [↑](#footnote-ref-46)
47. Proficient mismatch repair [↑](#footnote-ref-47)
48. Epstein-Barr Virus [↑](#footnote-ref-48)
49. Neutrophil-to-lymphocyte ratio [↑](#footnote-ref-49)
50. Lymphocyte-to-monocyte ratio [↑](#footnote-ref-50)
